# Psychometric Properties of the Short-Form Geriatric Depression Scale (GDS-SF) and Its Associated Factors among the Elderly in Bangladesh

**DOI:** 10.3390/ijerph19137935

**Published:** 2022-06-28

**Authors:** Naznin Sultana, Thao T. P. Nguyen, Ahmed Hossain, Md. Asaduzzaman, Minh H. Nguyen, Ishrat Jahan, Kien T. Nguyen, Tuyen Van Duong

**Affiliations:** 1 Department of Public Health, North South University, Dhaka 1229, Bangladesh; naznin.sultana28phn@gmail.com (N.S.); ahmed.hossain@northsouth.edu (A.H.); ishrat.mouri787@gmail.com (I.J.); 2 Health Personnel Training Institute, University of Medicine and Pharmacy, Hue University, Hue 49120, Vietnam; nguyenthiphuongthao@hueuni.edu.vn; 3 Global Health Institute, North South University, Dhaka 1229, Bangladesh; 4 Research and Development, Health Management BD Foundation, Cox’s Bazar 4700, Bangladesh; 5 Department of Public Health Nutrition, Primeasia University, Dhaka 1213, Bangladesh; asad.bdphn@gmail.com; 6 International Ph.D. Program in Medicine, College of Medicine, Taipei Medical University, Taipei 110-31, Taiwan; d142108015@tmu.edu.tw; 7 Department of Health Promotion, Faculty of Social and Behavioral Sciences, Hanoi University of Public Health, Hanoi 11910, Vietnam; 8 School of Nutrition and Health Sciences, Taipei Medical University, Taipei 110-31, Taiwan

**Keywords:** short-form geriatric depression scale, psychometric properties, reliability, validity, principal component analysis, peer support, satisfaction, the elderly, Bangladesh

## Abstract

Background: This study aimed to (1) evaluate the psychometric properties of a Comprehensive Short-Form Geriatric Depression Scale (GDS-SF) and (2) examine the associated factors of GDS-SF among the elderly. Methods: A cross-sectional study was conducted from November 2019 to April 2020 in Dhaka City Corporation, Bangladesh. Data of 377 elderly were collected, including socio-demographic characteristics, social supports, comorbidities, sleep behaviours, and depression (as measured by the GDS-SF). We used the principal component analysis, correlation analysis, and logistic regression analysis to validate GDS-SF, and explore its associations. Results: The GDS-SF was reliable and homogeneous with Cronbach’s alpha = 0.836, and McDonald’s Omega = 0.841, with no floor/ceiling effects. The questionnaire demonstrated a good construct validity with item-scale convergent validity and KMO measure of sampling adequacy (0.869 for the total sample, 0.838 for the community subsample, and 0.851 for the slum subsample). In the multivariate model, older people had a higher likelihood of moderate and severe depression (OR, 1.06; 95% CI, 1.00, 1.12; *p* = 0.048). The likelihood of having moderate and severe depression was lower in men (OR, 0.48; 95% CI, 0.28, 0.85; *p* = 0.011) and those satisfied with their children’s support (OR, 0.17; 95% CI, 0.08, 0.35; *p* < 0.001), compared with their counterparts, respectively. Conclusions: The GDS-SF is a reliable and valid survey tool for evaluating depression in Bangladeshi older adults. Age, gender, and satisfaction with children’s support were predictors of depression.

## 1. Introduction

The rate of the world’s ageing population is climbing sharply. The proportion of the world’s senior population is predicted to rise from 900 million to 2 billion between 2015 and 2050 [1]. Bangladesh is home to around 166 million people, of which the elderly population is 15 million, which is predicted to increase to 36 million by 2050 [2]. This rapidly ageing population is becoming more vulnerable to a broader range of non-communicable diseases and mental or neurological disorders [3]. The most common mental and neurological disorders in the elderly are dementia and depression, which affect approximately 5 and 7% of the world’s older population, respectively [4]. In 2019, the prevalence of depression in Bangladesh in those aged 18–99 years was about 6.7% [5].

Depression is a medical condition characterised by endless sadness, discouragement, and feelings of worthlessness. It may be accompanied by decreased energy and attention, insomnia (sleep issues), reduced appetite, weight loss, and physical discomfort [6]. The known risk factors for depression are being female [7], of older age [8], reduced coping abilities [9], increased morbidity, an impaired level of functioning [10], grief [8], and loss of cognition [11]. Depression in later life has a significant impact on older persons’ quality of life and functioning abilities [12]. Moreover, research has demonstrated that depression in later life is often under-recognised and under-treated [12,13] due to a lack of knowledge and the perception of demands on medical care [3].

Because of the high prevalence of depression in the geriatric population, researchers have proposed routine screening for depressive symptoms in older adults [14,15]. A 30-item scale known as the Geriatric Depression Scale (GDS) was developed to assess depression in the elderly [16]. The 30-item GDS has been translated into several languages, for instance, Nepalese [17], Iranian [18], Korean [19], Chinese [20], and Filipino [21]. On the other hand, GDS-30 is a time-consuming screening tool for both doctors and patients. A short form of the GDS was created by Sheikh and Yesavage [22] for a simple and quick assessment. The short form of GDS comprises 15 items and is also effective for diagnosing depression in the aged. It is more straightforward, brief, and time-effective than GDS-30 [23,24]. The GDS-Short Form (GDS-SF) includes elements that deal with life satisfaction and happiness, excitement about new activities, curiosity, energy, hopelessness and helplessness, and fear of the future. As the shorter form assesses the subjective characteristics of depression, its primary purpose is to screen for depression rather than diagnostic classification [25].

Considering the good psychometric properties (Cronbach’s alpha = 0.94, test-retest r = 0.85), the GDS-SF has been validated and translated into different languages such as Iranian [18], Malay [23], Swedish [26], Spanish (Mexico) [27], Indian [28], and Japanese [29]. In addition, the GDS-SF was also translated and adapted to the Bangladeshi language [30]. In this context, depression in the Bangladeshi elderly was evaluated using the GDS-SF scale and Cronbach’s alpha was also satisfactory in those studies [31,32]. However, except for Cronbach’s alpha, other psychometric properties, such as the floor and ceiling effect, of the construct and convergent validity of the GDS-SF scale were not studied in Bangladesh. Therefore, we aimed to evaluate the psychometric properties and examine the associated factors of depression among the elderly in Dhaka, Bangladesh.

## 2. Materials and Methods

### 2.1. Study Design and Settings

A community-based cross-sectional study was conducted from November 2019 to April 2020 in Dhaka City Corporation, Bangladesh. Dhaka City Corporation (DCC) is divided into two parts, North- and South City Corporations. The Dhaka North City Corporation (DNCC) consists of 54 wards and 1639 slums, whereas the Dhaka South City Corporation (DSCC) contains 57 wards and 1755 slums [33]. As it was hard to access the sampling frame, the wards and slums were conveniently selected. Ward 43, 19 and the slums of ward 19 were selected from DNCC. Ward 34 and one slum of this ward were selected from DSCC.

### 2.2. Sampling and Sample Size

The sample size was calculated using the following Formula (1):

(1) $$n=\frac{Z^{2}pq}{d^{2}}$$

where *Z* = a critical value at a 95% confidence level (=1.96) and p is the prevalence of the interest variable. A previous study in the Meherpur District of Bangladesh revealed that the prevalence of depression in the elderly was 23.0% [34], *q* = 1 − *p* and the effect size is 0.05, as suggested for a cross-sectional design [35]. The minimal sample size for this study was 272. We collected data from 377 elderly, which was satisfactory for analysis.

### 2.3. Study Participants and Data Collection Procedure

According to the UN (United Nations), the cut-off value for the elderly is 60 years and above, referred to as older or elderly persons [36]. In Bangladesh, the cut-off point for old age is 60 [37]. Therefore, our study participants were people whose age is equal to or greater than 60 years, who lived in the Dhaka City Corporation, and who were retired. Those suffering from chronic systemic illnesses (cancer, stroke, and Alzheimer’s disease), who had cognitive impairments, or who had lost a relative within the last six months were excluded from the study.

A semi-structured questionnaire was used two collect the data from both DNCC and DSCC. Two enumerators were hired who have been experienced in the data collection field for more than 3 years. An expert from a mental health background and the principal investigator also participated in the data collection team. Enumerators went door-to-door in both communities and slums for data collection. The participants were informed about the purposes of the study and signed the consent forms before taking part in an interview. It took 20 min for each interview. The data collected were cleaned, coded, and analysed anonymously.

### 2.4. Instruments and Measurements

#### 2.4.1. Socio-Demographics

Socio-demographic indicators assessed included the place of living (community or slums), age, gender (women or men), marital status (married/single/divorced/widowed), educational level (no formal education; primary school; secondary school or higher), having children (yes or no), house owner (yes or no), and religious practice (yes or no). The previous employment status is categorised into housewife (have no income), own business, employee, and other (part-time job, day labour, maid, etc.)

#### 2.4.2. Comorbidity

Participants were asked whether they had been diagnosed by a doctor with one of the following diseases: diabetes, cardiovascular disease, respiratory illness, kidney diseases, bone diseases, and other diseases (liver disease, fever, thyroid-related problems).

#### 2.4.3. Sleep Duration and Social Support

Sleep-related information was collected, such as hours/day of sleep in the daytime and nighttime and whether participants took a sleeping pill or not (yes or no). Sleeping hours/day (daytime and nighttime) were summarised and classified into “Sleep adequacy” and “Sleep deprivation”. Sleep deprivation was assessed by an average number of hours of sleep in 24 h. Following the recommendation of sleep duration for adults, the variable of sleep duration was categorised as “less than 7 h” (sleep deprivation) and “7 h and more” (sleep adequacy) [38,39].

In terms of social support, we collected information regarding support from the surrounding people (yes or no), support from peers (yes or no), and satisfaction with children’s support (yes or no).

#### 2.4.4. Geriatric Depression

The Geriatric Depression Scale-Short form (GDS-SF) was developed by Sheikh and Yesavage [22] and used as a primary screening measure for depression in older adults. It consists of 15 questions with binary responses “yes” or “no.” When 10 of the 15 items are answered affirmatively, they indicate the presence of depression, whereas the others (question numbers 1, 5, 7, 11, 13) suggest depression when responded to negatively. The scale has a total score of 15, with depression levels defined as normal (scores 0–4), mild (scores 5–8), moderate (scores 9–11), and severe (scores 12–15) [40].

The translation of the GDS-SF into Bangla included forward translation and backward translation [41]. *Forward translation:* The Bangla version was independently translated by two different bilingual translators (an academician and a mental health expert), whose mother tongues were Bangla. A mental health expert conducted this translation to provide a more clinical perspective of equivalence and produce a more reliable translation from a measurement perspective. In order to emphasise the obscure meanings in the original questionnaire, the academician was supposed to provide a translation in the language used by the population. A single quality-assured translation was then produced by harmonising and synthesising both translations. *Backward translation:* After that, two different bilingual translators (as a quality control step) back-translated the concept into English, producing two back-translated versions of English. These translators were also fluent in both English and Bangla. To determine the validity of the questionnaire, the item content needed to be equivalent to that of the original version.

### 2.5. Ethical Approval

The institutional ethical review committee of the North South University in Bangladesh approved the study (ref: 2021/OR-NSU/IRB/0803). Before participating, all individuals were asked for their permission and completed consent papers.

### 2.6. Statistical Analyses

Descriptive statistics were used to explore the distribution of different study variables. Mean, standard deviation (SD), percentage (%), median, interquartile range (IQR), minimum, or maximum were reported. The Kruskal–Wallis H test and one-way ANOVA test were used appropriately.

*Floor and ceiling effects:* A percentage of 15% or less at the floor or ceiling effects was considered a significant effect [42].

*Reliability*: Item analysis of each questionnaire involved estimating item difficulties, discrimination, and internal consistency. Item difficulties were determined by calculating the mean total score of each item. A mean score below the midpoint (0.5 on a scale ranging between 0 and 1 for the GDS-SF) was interpreted as a generally difficult aspect of scale in a consultation. Discrimination (i.e., how efficient the items individually contribute to the scale) was assessed by computing corrected item–total correlations. Internal consistency reliability of both scales as a whole was assessed using Cronbach’s α and McDonald’s Omega [43]. Internal consistency was considered good if 0.8 ≤ Cronbach’s α/McDonald’s Omega < 0.9 and excellent if Cronbach’s α/McDonald’s Omega > 0.9 [44,45,46].

*Construct validity*: To examine the construct of the GDS-SF, principal component analysis (PCA) was conducted. The Kaiser–Meyer–Olkin Measure of Sampling Adequacy (KMO) was used to determine the suitability of the data for component analyses. The recommended KMO value was ≥ 0.5 and values between 0.5 and 0.7 are mediocre, values between 0.7 and 0.8 are good, values between 0.8 and 0.9 are great, and values above 0.9 are superb [47]. Bartlett’s Test of Sphericity value was less than 0.05 [48].

*Convergent validity*: Spearman’s correlation coefficient (r_s_) of the total GDS-SF scores and its 15 items (item–total correlations) were checked. Correlation coefficients (r_s_) were considered very weak if < 0.20, weak if 0.20–0.39, moderate if 0.40–0.59, strong if ≥ 0.6–0.79, and very strong if 0.8–1 [49].

*Known-group validity* was also assessed through a comparison of the proportion of mild, moderate and severe depression vs. normal groups between groups with a different gender (male vs. female) and satisfaction with children’s support (yes vs. no).

*Associated factors of GDS-SF*: The simple and multiple ordinal logistic regression analyses were utilised to examine the factors associated with depression levels. The factors included in the multivariate model were those associated with depression at *p* < 0.20 in the bivariate model [50]. In order to avoid multicollinearity, Spearman’s correlation coefficient test was used to check associations between independent variables. If independent variables correlated with one another at Spearman’s correlation coefficient (rho) ≥ 0.3, one representative independent variable was selected for the multiple regression model.

A *p*-value of < 0.05 was considered statistically significant for all analyses.

The Feldt’s test was carried out in R using the ‘cocron’ command [51], and all other statistical analyses were performed using SPSS 25.0 (IBM Corp.: Armonk, NY, USA) [52].

## 3. Results

### 3.1. Participants’ Characteristics

In Table 1, the average age of the study population was 65.7 ± 5.1 years. Out of all participants, 29.2% lived in slums, 59.4% were men, 76.9% got married, 94.7% had at least one child, 33.2% finished secondary school or higher education, 24.7% had no income, 92.8% had at least one comorbidity, 29.2% did not practice religious activities, 15.6% slept less than 7 h per day, and 35.3% took sleeping pills. In addition, the mean score of the GDS-SF was 7.31 ± 3.98. Regarding social support, 70.6% received support from surrounding people in an emergency, 63.4% received support from peers, and 75.9% were satisfied with children’s support.

The depression levels (normal, mild, and moderate and severe) were statistically varied by different categories of living place, sex, marital status, education, owning a house, practising religious activities, having children (not including grandchildren), support from surrounding people in an emergency, support from their peers, satisfaction about the children’s support, and taking sleeping pills (*p* < 0.05; Table 1).

### 3.2. Psychometric Properties of GDS-SF

#### 3.2.1. Reliability (Internal Consistency)

The scale reliability (McDonald’s Omega and Cronbach’s α), difficulty (mean, SD), and discrimination of measurement for each of the items were presented in Table 2. There were seven items that had means lower than the mid-point of 0.5 (items 1, 3, 6, 8, 11, 12, and 14). The item difficulty values ranged from 0.23 (item 1) to 0.79 (item 2). The corrected item–total correlations did not meet the threshold of >0.70 for all items. The overall internal consistency reliability of GDS-SF was good (Cronbach’s α = 0.836; McDonald’s Omega = 0.841). With respect to Cronbach’s α, there was a statistically significant difference between the community and slum subsamples (0.816 vs. 0.883; *p* = 0.011).

#### 3.2.2. Construct Validity

A principal component analysis (PCA) was conducted on the 15 items of the GDS-SF with orthogonal rotation (varimax) (Table 2) and resulted in three main factors with an eigenvalue > 1 for all three samples studied (total sample (n = 377), the community subsample (n = 267), and the slum subsample (n = 110)). The scree plot also indicated that three factors were responsible for 52.42% of the variance in the GDS-SF in the total sample and 49.35% in the community subsample, whereas four factors explained the variance (68.34%) in the slum subsample. The KMO measure verified ‘great’ sampling adequacy (0.869 for the total sample, 0.838 for the community subsample, and 0.851 for the slum subsample). Bartlett’s test for sphericity confirmed the statistical relevance of the models (*p* < 0.001).

#### 3.2.3. Convergent Validity

The Spearman correlations between each item and the scale ranged from 0.225 to 0.732 (Table 3), in which three items (2, 9, and 10) had weak correlations with the GDS-SF total scores and seven items (1, 4, 6, 7, 11, 13, and 15) had moderate correlations; the remaining five items (3, 5, 8, 12, and 14) had strong correlations.

#### 3.2.4. Known-Group Validity

The results from the ANOVA test in Table 1 showed that the GDS-SF scores were significantly different between males and females (*p* < 0.05), and those who felt unsatisfied with their children’s support were more likely to experience depression compared to their counterparts (*p* < 0.05).

### 3.3. Factors Associated with GDS-SF

Table 4 shows the results of the simple and multiple ordinal logistic regression models for examining the predictors of mild depression (GDS-SF scores 5–8) and moderate and severe depression (GDS-SF scores 9–15).

In the bivariate model of the comparisons between individuals with mild depression and those with no depression, the odds of mild depression were significantly higher in people taking sleeping pills and who practised religious activities compared to their counterparts (*p* < 0.05); the likelihood of mild depression was significantly lower in people who were business owners or who were previously part-time employees compared to their counterparts (*p* < 0.05).

With the bivariate model of the comparisons between people with moderate and severe depression and those with no depression, the odds of moderate and severe depression were significantly lower in married males who attained primary school or higher, who were business owners or full-time employees or previously part-time employees, who received support from surrounding people and their peers, and who felt unsatisfied about their children’s support compared to their counterparts (*p* < 0.05).

We checked the correlations among the independent variables in Supplementary Table S1. There were moderate correlations between living place and education (rho = −0.32), owning a house (rho = 0.49), religious practice (rho = 0.33), and taking sleeping pills (rho = 0.32); then, living place was selected for the multiple ordinal logistic regression model. We also found sex moderately correlated with marital status (rho = 0.37) and occupation (rho = 0.37), so sex was selected for the multivariate model. There were moderate correlations between satisfaction with children’s support and having children (rho = 0.42), receiving support from surrounding people (rho = 0.37), and receiving support from peers (rho = 0.31); then, satisfaction with children’s support was selected for the multiple ordinal logistic regression model. Moreover, the factors included in the multivariate model were those that had an association with depression at *p* < 0.20 in the bivariate model. As a result, there were no significant predictors in the multivariate model of the comparisons between people with mild depression and those with no depression. In the multivariate model of the comparisons between people with moderate and severe depression and those with no depression, age was a significant positive predictor (OR, 1.06; 95% CI, 1.00, 1.12; *p* = 0.048) indicating that persons who were older were more likely to have moderate and severe depression. Males had lower odds of moderate and severe depression compared to females (OR, 0.48; 95% CI, 0.28, 0.85; *p* = 0.011). Participants who were satisfied with their children’s support (OR, 0.17; 95% CI, 0.08, 0.35; *p* <0.001) were less likely to have moderate and severe depressive symptoms.

## 4. Discussion

This study evaluates the diagnostic value of the GDS-SF in Dhaka, Bangladesh, by assessing its reliability, floor and ceiling effect, construct, known-group, and convergent validities. The results showed that the GDS-SF was a valid and reliable tool to measure depression among the elderly in Bangladesh, with adequate item-scale convergent validity, evidence of known-group validity, no apparent floor/ceiling effects, and high levels of internal consistency reliability. Of the two reliability analyses, both Cronbach’s alpha and McDonald’s Omega were performed. The GDS-SF showed good internal consistency (Cronbach’s alpha = 0.836) in the current study population, which was slightly lower than previous studies conducted in Iran (Cronbach’s alpha = 0.90) [18], Mexico (Cronbach’s alpha = 0.84) [27], and India (Cronbach’s alpha = 0.87) [28], and higher than community-living older Asian adults (Cronbach’s alpha = 0.80) [53]. We also performed the McDonald’s Omega for reliability analysis for the first time. According to methodologists, Cronbach’s alpha is not an optimum measure of reliability compared to McDonald’s Omega’s more generic counterpart. The reason for the widespread adoption of Cronbach’s alpha is that the computation of Omega (ω) is not available in many popular statistics programs and requires item loading from a confirmatory factor analysis (CFA) [54]. Different studies showed that it is one of the best alternatives for estimating reliability [45,55]. Omega offers the advantage of taking into account the strength of the link between items and constructs and item-specific measurement errors compared to Cronbach’s alpha. As a result, Omega delivers more realistic estimations of genuine scale reliability. McDonald’s Omega for this study was also satisfactory at 0.84. Therefore, the GDS-SF was shown to have satisfactory construct validity with a good model–data fit. In addition, the GDS-SF was shown to be a valid survey tool to measure depression in Bangladeshi older adults, including the community and slum subsamples, with the KMO measure verifying ‘great’ sampling adequacy. Consequently, the need for a simple screening tool such as the GDS-SF that helps identify depressive symptoms in Bangladeshi older adults is underlined.

In this study, the distributions of age and gender were nearly identical to previous studies conducted among the elderly in rural Bangladesh [32,56]. However, the number of male participants was higher due to the non-probability sampling method and inclusion criteria. Moreover, the proportion of moderate and severe depressive symptoms among females was higher than in males. This finding is consistent with other studies, such as Rahman et al. in 2019, which found that females had a higher proportion of severe depression (16.7%) than males (9.9%) [57]. Another study conducted by Hossain et al. indicated that the prevalence of depression in females and males was 47.3% and 39.8%, respectively [45]. Moreover, in India, the prevalence of depression was significantly higher among females (52%) in comparison to males (20%) [58]. In connection with this, the highest prevalence of depression among females was also recorded in Egypt [59]. Previous research found that females had a higher average life expectancy than males, and social, economic, and psychological factors may influence females to be more depressed than males [60,61].

Similarly, our study also found that older people had higher moderate and severe depression compared to the younger elderly. The findings were consistent with other studies, which found that moderate and severe depressive levels were higher among elders in various nations and periods [57,62,63].

Social factors, including social support from family members or friends, were found to protect older adults from the risks of depression [64]. Our study found that participants who received support from surrounding people, peers, and children in case of emergency were less likely to suffer from depression than their counterparts. In both the bivariate and multivariate ordinal regression models, satisfaction with children’s support was associated with a lower likelihood of moderate and severe depression. This finding is consistent with a study conducted among elderly Bangladeshi people in 2019 [65]. Adult children, particularly boys, are seen as the primary source of security and economic assistance for their parents in Bangladesh, particularly during disasters, sickness, and old age [66]. Bangladesh is one of the Asian countries with a long history of cultural and religious traditions dedicated to caring for the elderly. However, dramatic socioeconomic and demographic changes, widespread poverty, shifting social and religious norms, Western culture’s influence, and other factors have weakened the traditional extended family and community care structure. As a result, the majority of the elderly are facing difficulties regarding inadequate financial support, different types of diseases, and the absence of proper health and medical facilities, and they are often neglected [67]. Our findings also emphasised an urgent need for greater awareness of depression among family members and the community at large.

There are certain limitations to the current study. First, we could not use the probability sampling method and the inclusion criteria were retired people. As a result, the collected sample may not fully represent the general Bangladeshi elderly population. Second, we did not use a validated questionnaire to evaluate social support, which may affect the results; future research should use reliable instruments to assess these factors. Thirdly, the discriminant validity of the GDS-SF cannot be measured due to the fact that no other related measures were included in this study. Forth, the test–retest reliability was not evaluated as there were limited resources for collecting the data twice. Finally, this study could not provide cause-and-effect relationships and the GDF-SF‘s responsiveness due to the cross-sectional design, so future longitudinal studies with larger samples are suggested to confirm these findings.

## 5. Conclusions

The GDS-SF was shown to be a reliable and valid survey tool to measure depression in Bangladeshi older adults. Age, gender, and satisfaction with children’s support were predictors of depression. The findings could provide evidence for effective public health interventions to reduce depression among the elderly in Bangladesh.

## Figures and Tables

**Table 1:** Participants’ characteristics and depression levels (n = 377).

Variables	Total (n = 377)	Depression Levels by GDS-SF Scores			
		Normal (n = 100)	Mild (n = 130)	Moderate and Severe (n = 147)	*p*-Value *
	n (%)	n (%)	n (%)	n (%)	
**Living place**					**0.026**
Communities	267 (70.8)	69 (25.8)	103 (38.6)	95 (35.6)	
Slums	110 (29.2)	31 (28.2)	27 (24.5)	52 (47.3)	
**Age** (years), mean ± SD	65.7 ± 5.1	65.1 ± 4.9	65.4 ± 5.3	66.3 ± 5.1	0.156
**Gender**					**0.033**
Women	153 (40.6)	31 (20.3)	52 (34.0)	70 (45.8)	
Men	224 (59.4)	69 (30.8)	78 (34.8)	77 (34.4)	
**Marital status**					**0.014**
Single/Divorced/Widowed	87 (23.1)	14 (16.1)	29 (33.3)	44 (50.6)	
Married	290 (76.9)	86 (29.7)	101 (34.8)	103 (35.5)	
**Highest level of education**					**0.008**
No formal education	125 (33.2)	27 (21.6)	33 (26.4)	65 (52.0)	
Primary school	127 (33.7)	35 (27.6)	51 (40.2)	41 (32.3)	
Secondary school or higher	125 (33.2)	38 (30.4)	46 (36.8)	41 (32.8)	
**Previous occupation**					0.072
Housewife	93 (24.7)	14 (15.1)	35 (37.6)	44 (47.3)	
Own business	80 (21.2)	26 (32.5)	23 (28.8)	31 (38.8)	
Employee	104 (27.6)	29 (27.9)	38 (36.5)	37 (35.6)	
Other (part-time job, etc.)	100 (26.5)	31 (31.0)	34 (34.0)	35 (35.0)	
**House owner**					**0.019**
No	175 (46.4)	48 (27.4)	48 (27.4)	79 (45.1)	
Yes	202 (53.6)	52 (25.7)	82 (40.6)	68 (33.7)	
**Religious practice**					**0.034**
No	110 (29.2)	34 (30.9)	27 (24.5)	49 (44.5)	
Yes	267 (70.8)	66 (24.7)	103 (38.6)	98 (36.7)	
**Comorbidity**					0.094
None	27 (7.2)	9 (33.3)	9 (33.3)	9 (33.3)	
1	157 (41.6)	49 (31.2)	48 (30.6)	60 (38.2)	
2+	193 (51.2)	42 (21.8)	73 (37.8)	78 (40.4)	
**Having children**					**0.043**
No	20 (5.3)	4 (20.0)	3 (15.0)	13 (65.0)	
Yes	357 (94.7)	96 (26.9)	127 (35.6)	134 (37.5)	
**Receiving support from surrounding people in an emergency**					**<0.001**
No	111 (29.4)	11 (9.9)	19 (17.1)	81 (73.0)	
Yes	266 (70.6)	89 (33.5)	111 (41.7)	66 (24.8)	
**Receiving support from peers**					**<0.001**
No	138 (36.6)	15 (10.9)	25 (18.1)	98 (71.0)	
Yes	239 (63.4)	85 (35.6)	105 (43.9)	49 (20.5)	
Satisfaction with children’s support **					**<0.001**
No	91 (24.1)	10 (11.0)	22 (24.2)	59 (64.8)	
Yes	286 (75.9)	90 (31.5)	108 (37.8)	88 (30.8)	
**Sleep duration**					0.643
Sleep deprivation	59 (15.6)	16 (27.1)	23 (39.0)	20 (33.9)	
Sleep adequacy	318 (84.4)	84 (26.4)	107 (33.6)	127 (39.9)	
**Taking sleeping pills**					**0.041**
No	244 (64.7)	70 (28.7)	73 (29.9)	101 (41.4)	
Yes	133 (35.3)	30 (22.6)	57 (42.9)	46 (34.6)	
**GDS-SF,** mean (SD)	7.31 ± 3.98				
Median (IQR)	7.0 (4.0, 11.0)				

Abbreviations: GDS-SF, the Geriatric Depression Scale-Short Form; SD, standard deviation; IQR, interquartile range; * the results of Kruskal–Wallis H test and ANOVA test, appropriately; ** n = 375, since 20 respondents (5.3% of total sample) who had no children answered unknown.

**Table 2:** The GDS-SF items and scale characteristics (internal consistency, construct validity).

Items	Ceiling Effect n (%)	Floor Effectn (%)	Difficulty ^a^ (Mean ± SD)	Discrimination (Corrected Item-Total Correlation)	Cronbach’s α if the Item Deleted	McDonalds’s Omega	KMO	Bartlett’s Test
**Total sample (n = 377)**								
Item 1			0.23 ± 0.42	0.495	0.824			
Item 2			0.79 ± 0.40	0.275	0.836			
Item 3			0.41 ± 0.49	0.568	0.819			
Item 4			0.59 ± 0.49	0.492	0.824			
Item 5			0.55 ± 0.50	0.537	0.821			
Item 6			0.30 ± 0.46	0.324	0.834			
Item 7			0.51 ± 0.50	0.485	0.824			
Item 8			0.41 ± 0.49	0.642	0.814			
Item 9			0.52 ± 0.50	0.110	0.847			
Item 10			0.55 ± 0.50	0.295	0.836			
Item 11			0.37 ± 0.48	0.466	0.826			
Item 12			0.44 ± 0.50	0.573	0.819			
Item 13			0.61 ± 0.49	0.051	0.823			
Item 14			0.41 ± 0.49	0.656	0.813			
Item 15			0.61 ± 0.49	0.471	0.825			
GDS-SF scores (0–15)	3 (0.8)	7 (1.9)	7.31 (3.98)		0.836	0.841	0.869	χ2 = 1645.35 (df = 105), *p* < 0.001
**Community subsample (n = 267)**								
Item 1			0.21 ± 0.41	0.457	0.805			
Item 2			0.72 ± 0.45	0.316	0.813			
Item 3			0.39 ± 0.49	0.505	0.800			
Item 4			0.59 ± 0.49	0.430	0.806			
Item 5			0.52 ± 0.50	0.483	0.802			
Item 6			0.34 ± 0.48	0.382	0.809			
Item 7			0.47 ± 0.50	0.428	0.806			
Item 8			0.39 ± 0.49	0.598	0.794			
Item 9			0.58 ± 0.50	0.192	0.822			
Item 10			0.63 ± 0.48	0.352	0.811			
Item 11			0.27 ± 0.45	0.373	0.809			
Item 12			0.43 ± 0.50	0.501	0.801			
Item 13			0.59 ± 0.49	0.417	0.807			
Item 14			0.36 ± 0.48	0.615	0.793			
Item 15			0.60 ± 0.49	0.414	0.807			
GDS-SF scores (0–15)	3 (1.1)	4 (1.5)	7.09 ± 3.81		0.816	0.817	0.838	χ2 = 980.660 (df = 105), *p* < 0.001
**Slum subsample (n = 110)**								
Item 1			0.28 ± 0.45	0.560	0.874			
Item 2			0.95 ± 0.21	0.158	0.886			
Item 3			0.45 ± 0.50	0.700	0.868			
Item 4			0.61 ± 0.48	0.631	0.871			
Item 5			0.65 ± 0.48	0.652	0.870			
Item 6			0.18 ± 0.39	0.276	0.885			
Item 7			0.61 ± 0.49	0.601	0.873			
Item 8			0.47 ± 0.50	0.729	0.866			
Item 9			0.39 ± 0.49	0.004	0.899			
Item 10			0.37 ± 0.49	0.300	0.886			
Item 11			0.59 ± 0.49	0.673	0.869			
Item 12			0.47 ± 0.50	0.729	0.866			
Item 13			0.66 ± 0.48	0.686	0.869			
Item 14			0.51 ± 0.50	0.736	0.866			
Item 15			0.64 ± 0.48	0.595	0.873			
GDS-SF scores (0–15)	0 (0)	3 (2.7)	7.84 ± 4.32		0.883	0.896	0.851	χ2 = 835.628 (df = 105), *p* < 0.001
**Community vs. Slum**			*p* = 0.117 ^b^		χ2 = 6.456 (df = 1), *p* = **0.011** ^c^			

Abbreviations: GDS-SF, the Geriatric Depression Scale-Short Form; KMO, Kaiser–Meyer–Olkin measure. Notes: **Item 1.** Are you really happy with your life? **Item 2.** Have you given up a lot of your hobbies and interests? **Item 3.** Do you have the feeling that your life is meaningless? **Item 4.** Do you get bored easily? **Item 5**. Do you seem to be in a pleasant mood most of the time? **Item 6.** Are you afraid that something bad is going to happen to you? **Item 7.** Do you feel happy most of the time? **Item 8.** Do you often feel helpless? **Item 9**. Do you prefer to stay at home rather than going out and doing new things? **Item 10.** Do you feel you have more problems with memory than most? **Item 11.** Do you think it is wonderful to be alive now? **Item 12**. Do you feel pretty worthless the way you are now? **Item 13.** Do you feel full of energy? **Item 14**. Do you feel that your situation is hopeless? **Item 15.** Do you think that most people are better off than you are? ^a^ Difficulty is measured on a 0–1 scale; ^b^ Independent-Samples T-Test; ^c^ Feldt’s test to compare 2 alpha-coefficients.

**Table 3:** Results of the GDS-SF inter-item correlations (n = 377).

Items	Item 1	Item 2	Item 3	Item 4	Item 5	Item 6	Item 7	Item 8	Item 9	Item 10	Item 11	Item 12	Item 13	Item 14	Item 15	GDS-SF
**Item** **1**	1.00															
**Item 2**	0.13	1.00														
**Item 3**	0.39	0.12	1.00													
**Item 4**	0.31	0.19	0.34	1.00												
**Item 5**	0.40	0.21	0.35	0.43	1.00											
**Item 6**	0.22	−0.14	0.34	0.17	0.24	1.00										
**Item 7**	0.37	0.18	0.28	0.36	0.56	0.22	1.00									
**Item 8**	0.36	0.18	0.51	0.32	0.30	0.35	0.33	1.00								
**Item 9**	0.01 *	0.04 *	0.08 *	0.06 *	−0.10 *	0.11	−0.10 *	0.11	1.00							
**Item 10**	0.06 *	0.05 *	0.22	0.13	0.06 *	0.21	0.04 *	0.28	0.35	1.00						
**Item 11**	0.33	0.28	0.29	0.22	0.36	0.08 *	0.43	0.31	−0.07 *	0.03 *	1.00					
**Item 12**	0.30	0.19	0.44	0.29	0.24	0.21	0.18	0.50	0.13	0.31	0.32	1.00				
**Item 13**	0.24	0.27	0.21	0.33	0.39	0.05 *	0.31	0.31	0.13	0.24	0.38	0.37	1.00			
**Item 14**	0.47	0.28	0.49	0.35	0.46	0.28	0.39	0.61	−0.05 *	0.11	0.46	0.45	0.30	1.00		
**Item 15**	0.21	0.18	0.27	0.28	0.24	0.15	0.21	0.39	0.17	0.21	0.19	0.44	0.30	0.38	1.00	
**GDS-SF**	0.56	0.37	0.65	0.58	0.62	0.42	0.57	0.72	0.23	0.40	0.56	0.66	0.59	0.73	0.56	1.00
**Item-scale convergent validity:** mean of r_s_ (range) = 0.55 (0.23–0.73)																

* *p* < 0.05; Note: Spearman correlation coefficients (r_s_): very weak if < 0.20, weak if 0.20–0.39, moderate if 0.40–0.59, strong if ≥ 0.6–0.79 and very strong if 0.8–1.0.

**Table 4:** Results of the simple and multiple ordinal logistic regression analyses predicting depression (n = 377).

	Mild Depression ^a^				Moderate and Severe Depression ^a^			
Variables	Bivariate Model		Multivariate Model		Bivariate Model		Multivariate Model	
	OR (95% CI)	*p*	OR (95% CI)	*p*	OR (95% CI)	*p*	OR (95% CI)	*p*
**Living place**								
Communities	1.0		1.0		1.0		1.0	
Slums	0.58 (0.32, 1.06)	0.078	0.79 (0.39, 1.60)	0.518	1.22 (0.71, 2.10)	0.475	1.24 (0.63, 2.43)	0.538
**Age** (1-year increment)	1.01 (0.96, 1.07)	0.692	1.01 (0.95, 1.07)	0.757	1.05 (0.99, 1.10)	0.083	1.06 (1.00, 1.12)	**0.048**
**Gender**								
Women	1.0		1.0		1.0		1.0	
Men	0.67 (0.39, 1.17)	0.160	0.67 (0.38, 1.16)	0.153	0.49 (0.29, 0.84)	**0.010**	0.48 (0.28, 0.85)	**0.011**
**Marital status**								
Single/Divorced/Widowed	1.0				1.0			
Married	0.57 (0.28, 1.14)	0.112			0.38 (0.20, 0.74)	**0.005**		
**Highest level of education**								
No formal education	1.0				1.0			
Primary school	1.19 (0.61, 2.32)	0.605			0.49 (0.26, 0.92)	**0.026**		
Secondary school or higher	0.99 (0.51, 1.93)	0.977			0.45 (0.24, 0.84)	**0.012**		
**Previous occupation**								
Housewife	1.0				1.0			
Own business	0.35 (0.15, 0.82)	**0.015**			0.38 (0.17, 0.84)	**0.017**		
Employee	0.52 (0.24, 1.15)	0.107			0.41 (0.19, 0.88)	**0.022**		
Other (part-time job, etc.)	0.44 (0.20, 0.96)	**0.040**			0.36 (0.17, 0.78)	**0.009**		
**House owner**								
No	1.0		1.0		1.0		1.0	
Yes	1.58 (0.93, 2.68)	0.092	1.30 (0.70, 2.41)	0.406	0.80 (0.48, 1.32)	0.376	0.91 (0.49, 1.70)	0.767
**Religious practice**								
No	1.0		1.0		1.0		1.0	
Yes	1.97 (1.09, 3.55)	**0.025**	1.76 (0.93, 3.30)	0.080	1.03 (0.60, 1.76)	0.913	1.15 (0.63, 2.13)	0.646
**Comorbidity**								
None	1.0				1.0			
1	0.98 (0.36, 2.68)	0.968			1.22 (0.45, 3.32)	0.691		
2+	1.74 (0.64, 4.72)	0.278			1.86 (0.69, 5.03)	0.224		
**Having children**								
No	1.0				1.0			
Yes	1.76 (0.39, 8.07)	0.464			0.43 (0.14, 1.36)	0.150		
**Receiving support from surrounding people**								
No	1.0				1.0			
Yes	0.72 (0.34, 1.60)	0.421			0.10 (0.05, 0.20)	**<0.001**		
**Receiving support from peers**								
No	1.0				1.0			
Yes	0.74 (0.37, 1.49)	0.402			0.09 (0.05, 0.17)	**<0.001**		
**Satisfaction with children’s support**								
No	1.0		1.0		1.0		1.0	
Yes	0.55 (0.25, 1.21)	0.137	0.53 (0.24, 1.20)	0.127	0.17 (0.08, 0.34)	**<0.001**	0.17 (0.08, 0.35)	**<0.001**
**Sleep duration**								
Sleep deprivation	1.0				1.0			
Sleep adequacy	0.89 (0.44, 1.78)	0.735			1.21 (0.59, 2.47)	0.601		
**Taking sleeping pills**								
No	1.0				1.0			
Yes	1.82 (1.05, 3.16)	**0.033**			1.06 (0.61, 1.85)	0.829		

Abbreviations: OR, odds ratio; CI, confidence interval; ^a^ Reference group is the normal group (GDS-SF scores 0–4).

## Data Availability

Data will be available on reasonable request from the corresponding author.

## Supplementary Materials

The following supporting information can be downloaded at: https://www.mdpi.com/article/10.3390/ijerph19137935/s1, Table S1: Spearman’s correlation coefficients (rho) of study independent variables.
