# Supplementary material for: Psychometric Properties of the Short-Form Geriatric Depression Scale (GDS-SF) and Its Associated Factors among the Elderly in Bangladesh
Source: Int J Environ Res Public Health. 2022 Jun 28;19(13):7935. doi: 10.3390/ijerph19137935 (PMC9266010; doi:10.3390/ijerph19137935)
Supplement: Supplementary file 1 [file ijerph-19-07935-s001.zip › ijerph-1704948-supplementary.pdf]

Psychometric properties of the Short-Form Geriatric Depression Scale (GDS-SF) and its associated factors among the elderly in Bangladesh

SUPPLEMENTARY TABLE S1 | Spearman's correlation coefficients (rho) of study independent variables.

	Living place	Age	Sex	Marital status	Education	Previous Occupation	House owner	Religious practice	Comorbidity	Having children	Surrounding people's support[a]	Peer's support [b]	Children's support satisfaction	Sleep duration	Taking sleeping pills
**Living place**	1.00														
**Age**	0.02	1.00													
**Sex**	-0.06	0.07	1.00												
**Marital status**	-0.13*	-0.05	0.37**	1.00											
**Education**	-0.32**	0.04	-0.04	-0.08	1.00										
**Previous Occupation**	0.06	-0.01	0.37**	0.21**	-0.05	1.00									
**House owner**	0.49**	0.01	-0.01	-0.07	-0.19**	-0.16**	1.00								
**Religious practice**	0.33**	0.08	0.04	-0.05	-0.14**	-0.07	0.28**	1.00							
**Comorbidity**	0.01	0.12*	0.06	0.07	-0.07	-0.07	0.13*	0.11*	1.00						
**Having children**	-0.07	0.05	0.05	0.29**	-0.05	-0.10	-0.08	-0.07	0.13*	1.00					
**Surrounding people's support[a]**	0.23**	-0.08	-0.02	0.07	-0.14**	0.04	0.19**	0.10	0.04	0.11*	1.00				
**Peer's support [b]**	0.17**	-0.05	0.05	0.16**	-0.16**	0.05	0.14**	0.14**	0.09	0.16**	0.63**	1.00			
**Children's support satisfaction**	0.05	0.05	0.06	0.13*	-0.13*	0.09	0.10	0.03	0.07	0.42**	0.37**	0.31**	1.00		

**Sleep duration**	-0.04	-0.03	0.06	0.01	0.12*	0.04	0.04	0.03	0.05	-0.04	0.06	-0.02	0.03	1.00	
**Taking sleeping pills**	0.32**	-0.03	0.01	-0.04	-0.09	-0.03	0.28**	0.14**	0.08	0.01	0.09	0.02	0.01	-0.02	1.00

**p*<0.05; ***p*<0.01.

[a] *Getting support from surrounding people;* [b] *Getting support from peers.*
